# In vitro high glucose increases apoptosis, decreases nerve outgrowth, and promotes survival of sympathetic pelvic neurons

**DOI:** 10.1093/sexmed/qfac009

**Published:** 2023-02-01

**Authors:** Wrenn D Pallas, Elena S Pak, Johanna L Hannan

**Affiliations:** Department of Physiology, Brody School of Medicine at East Carolina University, Greenville, NC, United States; Department of Physiology, Brody School of Medicine at East Carolina University, Greenville, NC, United States; Department of Physiology, Brody School of Medicine at East Carolina University, Greenville, NC, United States

**Keywords:** erectile dysfunction, diabetes mellitus, glucose, peripheral nerves, Schwann cells

## Abstract

**Background:**

Diabetes mellitus (DM) is a common cause of erectile dysfunction (ED), yet the molecular basis of DM neurogenic ED remains unknown.

**Aim:**

In this study we examined the impact of high glucose on survival and growth of primary cultured pelvic neurons in a rat model and assessed whether coculturing with healthy Schwann cells (SCs) can rescue pelvic neuron growth in patients with DM.

**Methods:**

Major pelvic ganglia (MPGs) from adult male Sprague Dawley rats (*n* = 8) were dissociated and plated on coverslips. Neurons were exposed to high glucose (45 mM) for 24 or 48 hours and compared to time-matched controls (25 mM). Neurons were stained for neuron-specific beta-tubulin, neuronal nitric oxide synthase, vesicular acetylcholine transferase, tyrosine hydroxylase, and TUNEL (terminal deoxynucleotidyl transferase-mediated dUTP-biotin nick-end labeling) assay. Schwann cells were dissociated from MPGs of healthy male Sprague Dawley rats (*n* = 4) and grown to confluence. Additional Sprague Dawley rats were made diabetic with streptozotocin (50 mg/kg, *n* = 4), and 5 weeks later MPGs were collected from these rats, dissociated, and cocultured on healthy SCs. Neurons and SCs were stained with beta-tubulin and S100.

**Outcomes:**

Length, branching, and survival of nitrergic, parasympathetic, and sympathetic neurons was assessed in neurons exposed to normal or high glucose concentrations, and neuron length was measured in neuron-SC coculture.

**Results:**

The total number of neurons and the length and number of branches were significantly decreased after 24 and 48 hours of high glucose (*P* < .05). The percentage of nitrergic neurons decreased 10% after 24 hours and 50% after 48 hours of high glucose (*P* < .05). After 24 hours of high glucose, cholinergic-positive neurons were unchanged; however, these neurons decreased 30% after 48 hours (*P* < .05). The proportion of sympathetic neurons increased 25% after 48 hours of high glucose (*P* < .05). At both timepoints, there was a 2-fold increase in the total apoptotic neurons with high glucose (*P* < .05). Neurite outgrowth recovered to control lengths after coculture of diabetic neurons with healthy SCs (*P* < .05).

**Clinical Translation:**

Glucose can be used as a tool to investigate the direct effects of DM on neuritogenesis. Our data suggest that an effective treatment for DM ED protects and repairs the penile neuronal supply.

**Strengths and Limitations:**

Exposing MPG neurons to high glucose offers a quick and, inexpensive proxy for DM-related conditions. A limitation of our study is that our model reflects type 1 DM, whereas clinically, most diabetic ED patients have type 2 DM.

**Conclusion:**

Culturing pelvic neurons in high glucose can be used as a tool to elucidate how to protect proerectile neurons from cell death and may lead to new therapeutic strategies for diabetic men suffering from ED.

## Introduction

Over half a billion people are currently living with diabetes mellitus (DM) worldwide.^1^ DM is a metabolic disease characterized by chronic elevated blood glucose levels. There are 2 main types of diabetes: type 1 DM (T1DM) is caused by the failure of pancreatic beta cells to produce insulin, and type 2 DM (T2DM) is due to insulin resistance coupled by a failure of the beta cells to compensate.^2^ In the United States, over 90% of diagnosed cases of diabetes are T2DM.^3^ DM is a common cause of erectile dysfunction (ED).^4^ The prevalence of ED is approximately 3.5-fold higher in diabetic men than in men without DM, and the incidence of both ED and DM has been increasing over the years.^5^ Additionally, diabetic men have increased severity of ED and reported a less satisfactory sex life and lower quality of life.

The penis is innervated by autonomic (parasympathetic and sympathetic) and somatic (sensory and motor) nerves which can be damaged by DM. Oral phosphodiesterase-5 (PDE5) inhibitors are a first-line therapy for the treatment of ED^6^; however, these drugs require at least partially intact neural pathways to be effective. Damage to the cavernous and penile nerves because of long-standing or untreated DM leads to ineffective treatment with PDE5 inhibitors.^7^ There is a need to develop treatments to improve erectile function in DM patients.

Experiments in animal models have enabled our understanding of the pathogenesis of ED secondary to DM, leading to the development of prevention strategies and novel treatments. The rat is the most common laboratory animal used for developing models of ED.^8^ Multiple diabetic rat models are available to study ED, but each has its limitations. Genetic models are available, but they are expensive. Injecting rats with streptozotocin (STZ) damages the insulin-producing beta cells of the pancreas, leading to T1DM. Previous work has demonstrated that male rats fed a high-fat diet and receiving 2 low-dose injections of STZ can be used to model lean T2DM-associated ED.^9^ However, the challenges of STZ-induced DM in the rat model include sustaining uniformity, suitability, reproducibility, and induction of DM with minimal animal lethality.^10^ These challenges prompted us to evaluate if exposing dissociated (major pelvic ganglia) MPG neurons to high glucose concentrations is a method that may provide a quick and inexpensive proxy for DM conditions that can be used to further understand DM-induced neuronal dysfunction.

The objective of the present study was to examine the effects of high glucose on MPG-dissociated neuron outgrowth, branching, and survival of parasympathetic and sympathetic neurons. In addition, we measured MPG neurite length from control and DM rats in the presence and absence of control Schwann Cells (SCs) to examine if neuritogenesis can be rescued by SCs.

## Methods

### Animals

Male Sprague Dawley rats (*n* = 20; aged 12-18 weeks; Charles River Laboratories, Wilmington, MA) were provided food and water ad libitum and housed in pairs (25°C with a 12-hour light/dark cycle). Eight rats were used for the dissociated culture experiments with varying levels of glucose. For coculture experiments, 4 rats were used as controls for dissociated MPG neuron collection, 4 rats were used for control SC MPG isolation and the remaining 4 rats were made diabetic with administration of streptozotocin (STZ; 50 mg/kg, dissolved in 0.1 M sodium citrate buffer, pH 4.5) for dissociated neuron collection. Diabetes was confirmed with a fasting blood glucose level greater than 200 mg/dL and MPG tissues were collected 5 weeks after initial injection. For MPG collection, rats were anesthetized and bilateral MPGs were carefully dissected and placed into Hibernate A medium (Thermo Fisher Scientific, Waltham, MA). All procedures were performed in accordance with the ethical standards of the Institutional Animal Care and Use Committee Guidelines and the NIH guide for the care and use of laboratory animals.

### Dissociated MPG neuron culture in normal or high glucose

As previously described, paired MPGs were digested in collagenase and dispase (Sigma Aldrich, St. Louis, MO, USA), dissociated, and plated onto glass coverslips coated with laminin and poly-L-ornithine (Sigma Aldrich).^11^ Paired MPGs from 1 rat were divided among 8-9 coverslips for staining in duplicate for each condition (*n* = 4/group). Dissociated MPG neurons were incubated in basal medium (10% fetal bovine serum and Neurobasal Plus medium; Thermo Fisher Scientific) in control (25 mM) or high glucose (45 mM) conditions and assessed after 24 or 48 hours. Neurobasal medium contains 25 mM glucose, which is required to support normal neurite survival and growth. Our high-glucose group contained an additional 20 mM of glucose (total 45 mM) which was previously shown to mimic diabetic conditions by increasing reactive oxygen species, neurite degeneration, and apoptosis in rat dorsal root ganglia neurons.^12^

### Immunofluorescence staining and imaging in dissociated neurons

After 24 or 48 hours, neurons were fixed with 4% paraformaldehyde and incubated at 4°C overnight with primary antibodies specific for neuron-specific class III beta-tubulin (TUJ1; 1:100 rabbit or 1:500 mouse; Sigma-Aldrich), neuronal nitric oxide synthase (nNOS; 1:50 mouse; Santa Cruz Biotechnology, Dallas, TX, USA), tyrosine hydroxylase (TH; 1:250 rabbit, Millipore Sigma, Burlington, MA, USA), and vesicular acetylcholine transporter (VAChT; 1:100 rabbit; Santa Cruz) and counterstained with 4′,6-diamidino-2-phenylindole (DAPI; 1:500; Thermo Fisher Scientific). Secondary antibodies (Alexa Fluor 488, Alexa Fluor 594; Invitrogen, Carlsbad, CA) were incubated at room temperature (1 hour). Neurons were viewed with an Olympus IX81 fluorescent microscope (IMT-2; Olympus, Tokyo, Japan), and images were captured at 100*×* magnification with a Nikon TE200 camera (Diagnostic Instruments, Sterling Heights, MI). From each image (15-30 images per coverslip, 20-50 neurons per rat), neurons with clearly visible neurites were chosen and the longest neurite from each neuron was measured manually using Image J Software (NIH, Bethesda, MD). Branching was assessed on the longest neurite within each high-powered microscopic field by counting the number of branches that were at least 2 times the diameter of the nuclear cell body. For evaluation of autonomic neurons, only neurons whose axons were costained positively for both TUJ1 and nNOS, VAChT or TH were counted. Autonomic neuron counts were normalized to total number of neurons per coverslip.

### TUNEL assay in dissociated neurons

Apoptosis was assessed using an in situ cell death detection kit (TUNEL assay, Roche Diagnostics, Risch-Rotkreuz, Switzerland) and counterstained with TUJ1 and DAPI. Images were captured as previously described and apoptotic neurons were manually counted based on TUNEL staining within the nucleus of TUJ1-positive cells.^11^^,^^13^ The number of apoptotic neurons wase normalized to the total number of neurons on each slide.

### Dissociated diabetic MPG neuron/SC coculture

For SC dissociation and isolation, paired MPGs (*n* = 4 rats) were excised, digested in collagenase/dispase/hyaluronidase, and dissociated by gentle trituration. Dissociated SCs were suspended in a SC medium containing Dulbecco’s Modified Eagle Medium with d-valine (HiMedia, West Chester, PA) as previously described.^11^ Once confluent, SCs were plated onto individual glass coverslips coated with laminin and poly-l-lysine, covered with SC medium, and again incubated to confluence for 24-48 hours. Dissociated neurons from control and diabetic rats were plated on top of the confluent SC and cocultured for 48 hours in a 50:50 mixture of neurobasal and SC media which was changed every 24 hours. After 48 hours, cocultured neurons and SCs were fixed and stained with primary antibodies specific for TUJ1 (1:100, rabbit; Sigma-Aldrich), GFAP (1:200 anti-mouse; BioLegend, San Diego, CA), or S100 (1:200; anti-chicken, MyBioSource, San Diego, CA) captured as previously described, and the longest neurite from each neuron was measured and counterstained with DAPI.

### Statistical analyses

The number of animals used per group was determined based on previous experiments.^11^^,^^13^^,^^14^ Data are expressed as a mean ± SEM. Control and high-glucose/diabetic groups were compared using a 1-way ANOVA with Tukey’s multiple comparison test or a Student t-test. The Benjamini, Krieger, and Yekutieli method was used to control the false-discovery rate. *P* values of <.05 were used as criteria for statistical significance (Prism 5.0, GraphPad, La Jolla, CA). All experiments were performed in duplicate and counted, measured, and analyzed by two blinded independent investigators.

## Results

### MPG neuron length, branching and survival

**Figure 1:**
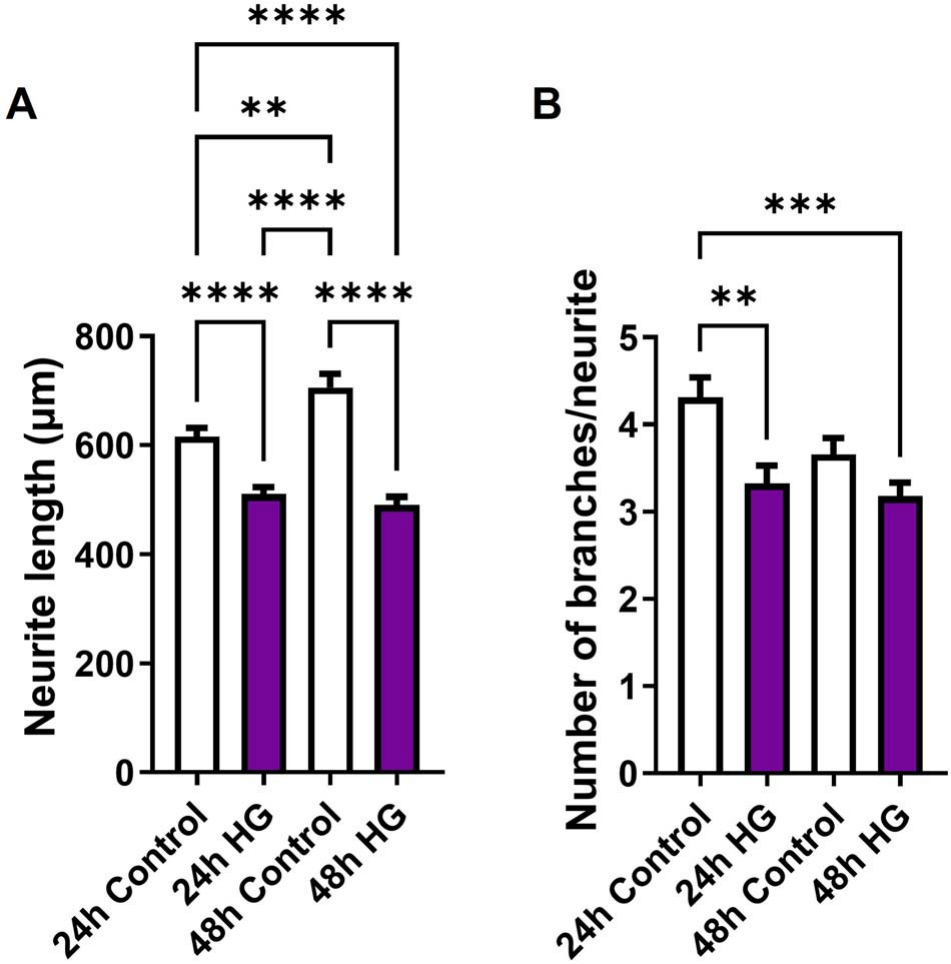
High glucose decreases neurite length and branching. Average neurite length (A) was significantly decreased after 24 and 48 hours of high glucose compared to control. High glucose significantly decreased the average number of branches per neurite (B) after 24 and 48 hours compared to control. Data are represented as mean ± SEM (^*^^*^P < .01, ^*^^*^^*^P < .001, ^*^^*^^*^^*^P < .0001, *n* = 4).

**Figure 2:**
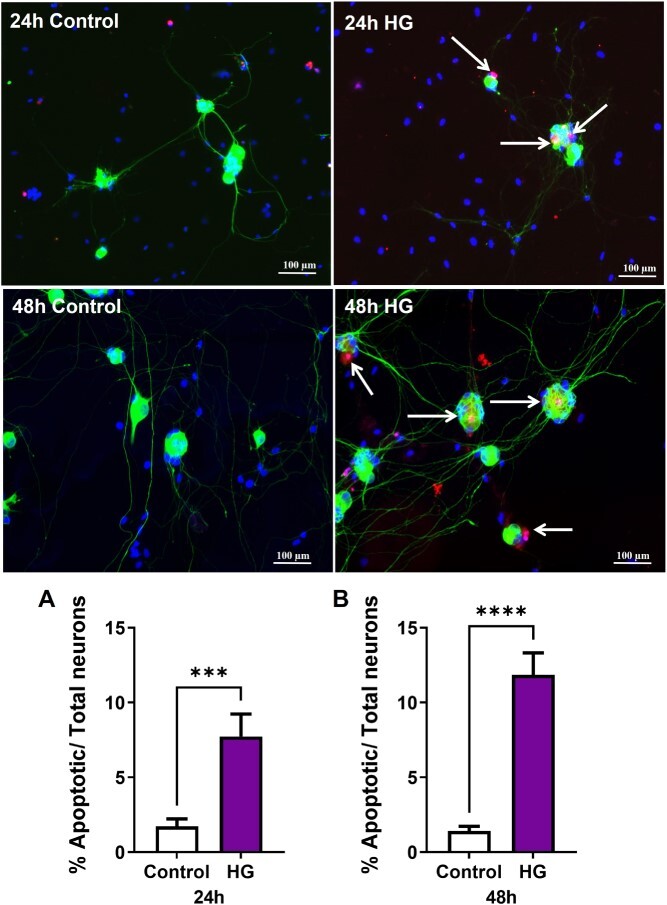
High glucose increases apoptotic cell death. Representative images of dissociated MPG neurons stained with TUNEL (red), βTubbIII (green), and DAPI (blue, ×10). The number of TUNEL-positive neurons increased significantly after (A) 24 and (B) 48 hours of high glucose compared to control. White arrows indicate TUNEL-positive neurons. Data are represented as mean ± SEM (^*^^*^^*^P < .001, ^*^^*^^*^^*^P < .0001, *n* = 4). DAPI, 4′,6-diamidino-2-phenylindole; MPG, major pelvic ganglion; TUNEL, terminal deoxynucleotidyl transferase-mediated dUTP-biotin nick-end labeling.

Dissociated MPG neurons were cultured in normal glucose or high glucose conditions and assessed after 24 or 48 hours. Over time, the neurons in normal glucose continued to elongate, whereas the neurons in high glucose did not grow any longer (Figure 1A). At both 24 and 48 hours, MPG neurons exposed to high glucose had shorter neurite lengths (Figure 1A). Additionally, at 24 hours MPG neurons exposed to high glucose had fewer branches compared to neurons in normal glucose (Figure 1B). We also assessed apoptosis using the TUNEL assay and found that high glucose exposure increased the number of neurons undergoing cell death by 348% and 732% at 24 and 48 hours, respectively (Figure 2).

### Nitrergic, parasympathetic, and sympathetic pelvic neuron populations

**Figure 3:**
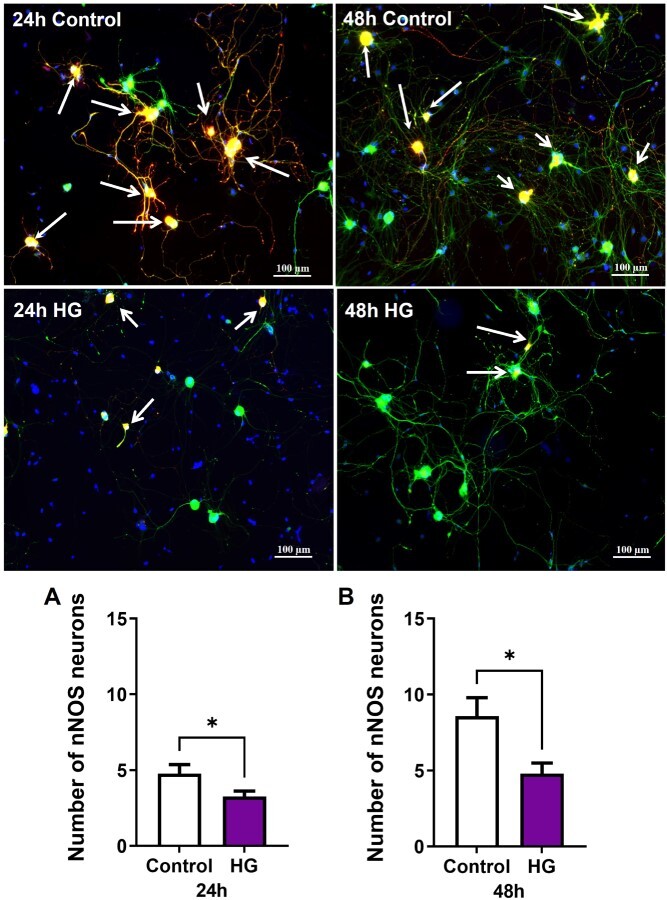
High glucose decreases nitrergic neurons. Representative images of dissociated MPG neurons stained with nNOS (red), βTubbIII (green), and DAPI (blue, x10). White arrows indicate nNOS positive neurons. The number of nitrergic neurons decreased significantly after (A) 24 and (B) 48 hours of high glucose compared to control. Neurons stained positive with nNOS or βTubbIII were counted from each high-powered field. Data are represented as mean ± SEM (^*^P < .05, *n* = 4). DAPI, 4′,6-diamidino-2-phenylindole; MPG, major pelvic ganglion; nNOS, neuronal isoform of nitric oxide synthase.

**Figure 4:**
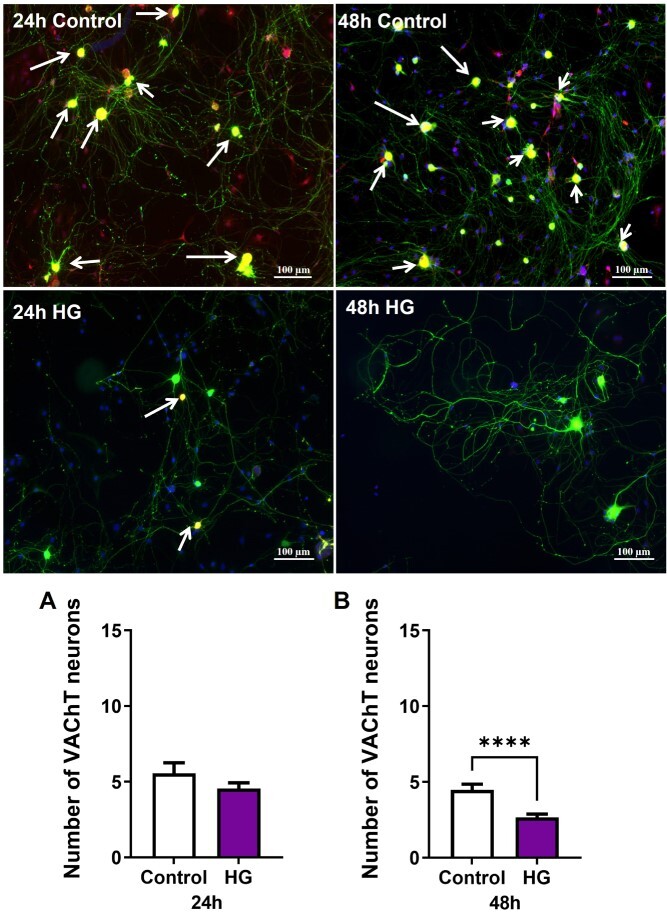
High glucose decreases cholinergic neurons. Representative images of dissociated MPG neurons stained with vesicular acetylcholine transporter (VAChT, red), βTubbIII (green), and DAPI (blue, ×10). The number of VAChT positive neurons increased significantly after 48 hours of high glucose compared to the control. White arrows indicate VAChT positive neurons. Data are represented as mean ± SEM (^*^^*^^*^^*^P < .0001, *n* = 4).

**Figure 5:**
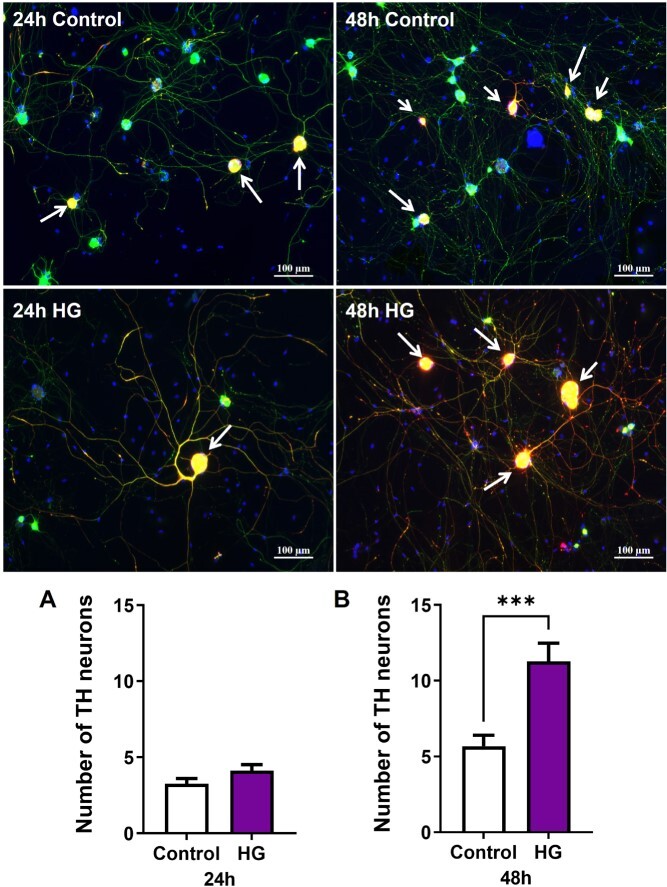
High glucose increases adrenergic neurons. Representative images of dissociated MPG neurons stained with tyrosine hydroxylase (TH, red), βTubbIII (green), and DAPI (blue, ×10). White arrows indicate TH-positive neurons. The number of TH-positive neurons increased significantly after 48 hours of high glucose compared to the control. Data are represented as mean ± SEM (^*^^*^^*^P < .001, *n* = 4). MPG, major pelvic ganglion.

Nitrergic, parasympathetic, and sympathetic nerve populations were identified by immunofluorescence staining with nNOS, VAChT, and TH, respectively. At both time points, high glucose exposure markedly decreased the relative number of nitrergic neurons (Figure 3). The number of neurons positive for VAChT was unchanged at 24 hours of high glucose and was significantly decreased after 48 hours in high glucose (Figure 4). At 24 hours, high glucose exposure did not change the number of neurons positive for TH. However, after 48 hours in high glucose, the number of TH neurons was significantly increased and double that of the control neurons (Figure 5).

### Dissociated diabetic neurons cocultured with healthy Schwann cells

**Figure 6:**
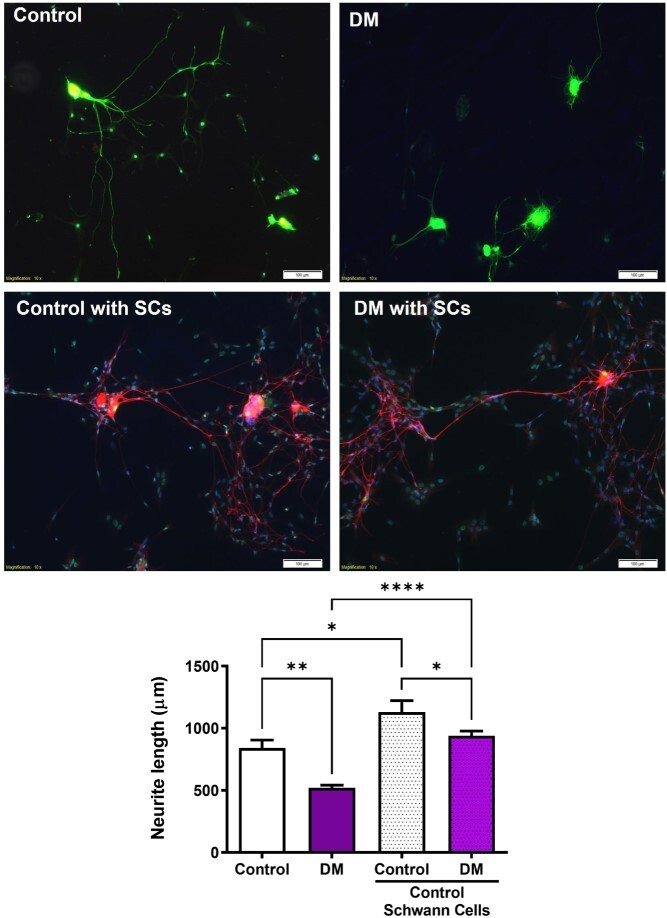
Schwann cells increase neurite length. Representative images of MPG neurons single were stained with βTubbIII (green) and DAPI (blue), and neurons cocultured with SCs were stained with GFAP (green), βTubbIII (red), and DAPI (blue, x10). DM neurites cocultured with SCs had significantly longer neurites than DM neurites without SCs. Data are represented as mean ± SEM (^*^P < .05, ^*^^*^P < .01, ^*^^*^^*^^*^P < .0001, *n* = 4). DAPI, 4′,6-diamidino-2-phenylindole; GFAP, glial fibrillary acidic protein; MPG, major pelvic ganglion.

We assessed neuritogenesis of pelvic neurons from diabetic rats by measuring neurite length. At the time of MPG collection, fasted blood glucose values were higher in diabetic rats (CON: 72 ± 6.4 vs DB: 245 ± 39.6 mg/dL; *P* < .001) and there was no change in body weight between groups (CON: 414 ± 15.1 vs DB: 385 ± 29.5 g; *P* = .133). Pelvic neurons from diabetic rats were 38% shorter in length than those of control rats (Figure 6). To assess whether coculture with healthy Schwann cells could rescue neurite growth, we cocultured healthy rat pelvic Schwann cells with control and diabetic pelvic neurons. Control neurons cocultured with Schwann cells grew an additional 34% compared to control neurons grown on their own. When diabetic neurons were cocultured with control Schwann cells, neurite length increased by 80% compared to diabetic neurons grown alone. The Schwann cells were able to rescue neuritogenesis, and the diabetic neurons cocultured with Schwann cells grew to the same length as the control neurons grown on their own.

## Discussion

In the present study, we confirmed that dissociated neurons exposed to high glucose mimic the diseased phenotype of neurons from DM rats and mice. In cultured pelvic neurons, high glucose exposure decreased neurite length and branching, decreased proerectile parasympathetic neurons, increased sympathetic neurons, and increased apoptosis. Additionally, to further investigate the interaction of neurons with the supporting cells present in their natural environment, we cocultured MPG neurons from STZ-induced DM rats with healthy SCs and analyzed neurite length. We found that DM reduced neurite length, but when cocultured with healthy SCs the length of DM neurons was rescued to the same length as that of the single cultured control neurons.

Generally, following injury, neuritogenesis begins with neurite sprouting, followed by elongation toward the target organ or tissue of innervation (ie, the penis), and last, trimming of unnecessary innervations to achieve functional recovery.^15^ Previous work by Matsui et al. demonstrated that culture of whole MPGs from T2DM rats resulted in a time-dependent decrease in MPG neurite length and sprouting.^16^ Similarly, Das et al. incubated MPGs from mice in high glucose to mimic diabetic neuropathy and reported a severe decrease in neurite outgrowth.^17^ In the present study, elongation and sprouting of MPG neurites were significantly decreased after 24 and 48 hours of high glucose, respectively. Our findings, along with those of previously reported studies, suggest that the impact of high glucose on neurons is similar to that of DM and therefore may be used to study the direct effects of DM on the ability of neurons to regenerate.

To initiate an erection, pelvic parasympathetic nitrergic nerves must release NO.^18^ Previous work by Cellek et al. demonstrated that neurogenic ED from DM was caused by the selective apoptotic cell death of nitrergic neurons in rat MPGs.^19^ Cellek et al. demonstrated that in rat MPGs 16 weeks after STZ-induced T1DM the relative number of nitrergic neurons decreased and the relative number of adrenergic neurons was unchanged. We obtained similar results when analyzing rat MPGs after 48 hours in high glucose. The relative number of parasympathetic (nitrergic and cholinergic) neurons decreased and the relative number of sympathetic (adrenergic) neurons increased. Our results suggest that high glucose disproportionately causes apoptotic cell death in both nitrergic and cholinergic neurons, resulting in a higher sympathetic tone. These findings support that DM disproportionately damages parasympathetic neurons and suggest that the pathogenesis of DM ED may be caused by the reduction of MPG nitrergic neurons.

The current proposed mechanism of neurogenic DM ED is that first, nitrergic nerves lose some of their nNOS protein content and function (this is reversible) and second, the nitrergic neurons undergo apoptosis (which is irreversible), suggesting that early treatment is necessary to prevent neurogenic ED in DM.^16^ Previous work by Nguyen et al. demonstrated that high glucose increased apoptosis and impaired tube formation in mouse cavernous endothelial cells and cavernous pericytes and decreased neurite sprouting from mouse MPGs.^20^ In the present study, we investigated how high glucose impacts apoptosis in rat MPGs and demonstrated that high glucose increased apoptotic cell death at both 24 and 48 hours. We hypothesize that high glucose disproportionately caused apoptotic cell death in nitrergic neurons, but a limitation of our TUNEL staining is that we did not costain with nitrergic or sympathetic specific markers, making it difficult to identify which type of neuron was undergoing apoptosis.

Following nerve injury, it is well documented that SCs play a crucial role in promoting recovery of the injured peripheral neurons by creating a neurotrophic environment to facilitate growth and survival by dedifferentiating into a repair phenotype to clear debris, release growth factors, and promote axon regeneration.^21–23^ To enhance our findings from our monocultures, we used our pelvic neuron–SC coculture system to model how DM impacts pelvic neuron axon outgrowth. Our findings support that Schwann cells were able to dedifferentiate into a repair phenotype and create a neurotrophic environment by releasing growth factors to promote axonal regeneration and survival. Thus, the presence of Schwann cells was able to mitigate the degenerative effects of diabetes and allow diabetic neurons cocultured with Schwann cells to grow neurites similar in length to healthy neurites grown alone. Previous work by Randolph et al. compared whole versus dissociated neuron cultures for studying the effect of prostatic radiation therapy (RT) on MPG neurite length.^14^ We showed opposing growth outcomes depending on the culture method used; in dissociated cultures, RT decreased neurite length, but in whole cultures, RT increased neurite length. The presence of SCs in the whole culture could explain this difference, which encouraged us to investigate the role of SCs during neuritogenesis using a neuron-SC coculture. Hyung et al. developed this coculture method of plating motor neurons on a feeder layer of SCs to gain a better understanding of the mechanisms of myelination and demyelination.^24^ Additionally, Pham et al. investigated diabetic neuropathy by incubating primary neurons from rat dorsal root ganglion cocultured with SCs in hyperglycemic conditions, which mimicked symptoms of diabetic neuropathy, such as impaired neurite extension and impaired myelination.^25^ In the present study, we cocultured dissociated healthy DM rat MPG neurons with or without healthy SCs, and measured changes in outgrowth to analyze the supporting role of SCs during neuritogenesis. We found that coculture with SCs increased neurite length of both control and DM neurons compared to their single-culture counterparts. Interestingly, DM neurons cocultured with SCs grew to the same length of the control neurons grown alone. Our results, along with previous reports, support the notion that SCs play an essential role in controlling axon outgrowth and repair of pelvic neurons.

A limitation of our coculture is that we did not assess apoptosis or the prevalence of different neuron types. Additionally, in our coculture experiments, all experiments were assessed in a rat model of T1DM, whereas clinically most diabetic ED patients have T2DM. In future studies we will isolate MPGs from experimental models of T2DM (ZDF rat) to further investigate the impact of hyperglycemia on pelvic nerve neuritogenesis. This will allow for more translational data applicable to the broader DM ED population.

## Conclusion

High glucose decreased neurite length and sprouting, decreased parasympathetic neurons, increased sympathetic neurons, and increased apoptosis in rat pelvic neurons. Additionally, SC coculture rescued DM neurites to the length of healthy neurites in monoculture. These results suggest that SCs provide a supportive environment for neurons that encourages growth and could be a potential therapeutic target for DM-induced ED. We conclude that high glucose can be used as a tool to investigate the direct effects of DM on neuritogenesis and suggest that an effective treatment for DM ED must protect and repair the penile neuronal supply. In future work we will examine the effect of DM on MPG neurite length in models of T2DM to better represent the clinical diabetic ED patient population.

## Funding

The production of the manuscript was supported by Brody School of Medicine start-up funds (J.L. Hannan). The funding sources have only supported the project financially and have not been involved in the design, production or handling of the intellectual content of the manuscript.

*Conflicts of interest:* None.
